# A Multilayered Control of the Human *Survival Motor Neuron* Gene Expression by Alu Elements

**DOI:** 10.3389/fmicb.2017.02252

**Published:** 2017-11-15

**Authors:** Eric W. Ottesen, Joonbae Seo, Natalia N. Singh, Ravindra N. Singh

**Affiliations:** Department of Biomedical Sciences, Iowa State University, Ames, IA, United States

**Keywords:** spinal muscular atrophy, SMA, survival motor neuron, SMN, SMN6B, Alu, exonization, transposable elements

## Abstract

Humans carry two nearly identical copies of *Survival Motor Neuron* gene: *SMN1* and *SMN2.* Mutations or deletions of *SMN1*, which codes for SMN, cause spinal muscular atrophy (SMA), a leading genetic disease associated with infant mortality. Aberrant expression or localization of SMN has been also implicated in other pathological conditions, including male infertility, inclusion body myositis, amyotrophic lateral sclerosis and osteoarthritis. *SMN2* fails to compensate for the loss of *SMN1* due to skipping of exon 7, leading to the production of SMNΔ7, an unstable protein. In addition, SMNΔ7 is less functional due to the lack of a critical C-terminus of the full-length SMN, a multifunctional protein. Alu elements are specific to primates and are generally found within protein coding genes. About 41% of the human *SMN* gene including promoter region is occupied by more than 60 Alu-like sequences. Here we discuss how such an abundance of Alu-like sequences may contribute toward SMA pathogenesis. We describe the likely impact of Alu elements on expression of SMN. We have recently identified a novel exon 6B, created by exonization of an Alu-element located within *SMN* intron 6. Irrespective of the exon 7 inclusion or skipping, transcripts harboring exon 6B code for the same SMN6B protein that has altered C-terminus compared to the full-length SMN. We have demonstrated that SMN6B is more stable than SMNΔ7 and likely functions similarly to the full-length SMN. We discuss the possible mechanism(s) of regulation of *SMN* exon 6B splicing and potential consequences of the generation of exon 6B-containing transcripts.

## Introduction

Transposable elements (TEs) including long and short interspersed elements (LINES and SINES) occupy ∼45% of human genome (Lander et al., 2001; Smit et al., 2015). The primate-specific Alu elements are the most abundant SINES totaling >1 million copies and accounting for ∼11% of the human genome (Lander et al., 2001; Hedges and Batzer, 2005; Deininger, 2011). Alu elements are ∼300 bp bipartite motifs derived from the 7SL RNA, which is one of the components of the protein signal recognition complex (Deininger et al., 2003). The spread of Alu elements started with the radiation of primates ∼65 million years ago (Mya) and peaked ∼40 Mya. Alu elements are broadly classified into three subfamilies J, S, and Y, with S and Y being the youngest and the only active subfamilies (Deininger, 2011). Insertion of Alu elements has played a significant role in primate evolution due to their drastic effect on chromatin remodeling and transcription, and the generation of novel exons (Gu et al., 2009; Antonaki et al., 2011; Cui et al., 2011; Su et al., 2014; Attig et al., 2016; Bouttier et al., 2016). TEs, including Alu elements, promote non-allelic homologous recombination (NAHR) and have caused and continue to contribute toward genomic instability (White et al., 2015; Wang et al., 2017). A recent genome-wide association study (GWAS) links Alu insertions to the high risk for many human diseases (Payer et al., 2017).

Alu-derived sequences affect various posttranscriptional steps including pre-mRNA splicing, mRNA stability, and translation (Lev-Maor et al., 2003; Aktaş et al., 2017; Elbarbary and Maquat, 2017). As per one estimate, ∼5% of alternative exons in humans are derived from Alu-like sequences (Sorek et al., 2002). Given the fact that transcripts carrying Alu exons harboring premature termination codon may skip detection due to nonsense-mediated decay (NMD) (Attig et al., 2016), this number could be an underestimation. Insertion of Alu-derived exons is generally suppressed by hnRNP C, which blocks recognition of the 3′ splice site (3′ss) by competing with the splicing factor U2AF65 (Zarnack et al., 2013). Inverted Alu repeats facilitate production of circular RNAs (circRNAs) due to their ability to loop-out sequences via stable double-stranded RNA structures (Liang and Wilusz, 2014; Wilusz, 2015). Depletion of DHX9, an RNA helicase that resolves the double-stranded RNA structures, was recently shown to enhance Alu-induced RNA processing defects including aberrant pre-mRNA splicing and circRNA production from transcripts harboring Alu repeats (Aktaş et al., 2017). In some instances, the stability of the Alu-derived transcripts is regulated by Adenosine Deaminase Acting on RNAs (ADARs) and the unmodified Alu-containing transcripts are degraded by Staufen-mediated RNA decay (SMD) (Elbarbary and Maquat, 2017). Consistently, a recent report suggests that the accelerated nuclear export of ADARs under stress-associated conditions leads to an enhanced stabilization of critical mRNAs harboring Alu repeats (Sakurai et al., 2017).

**FIGURE 1:**
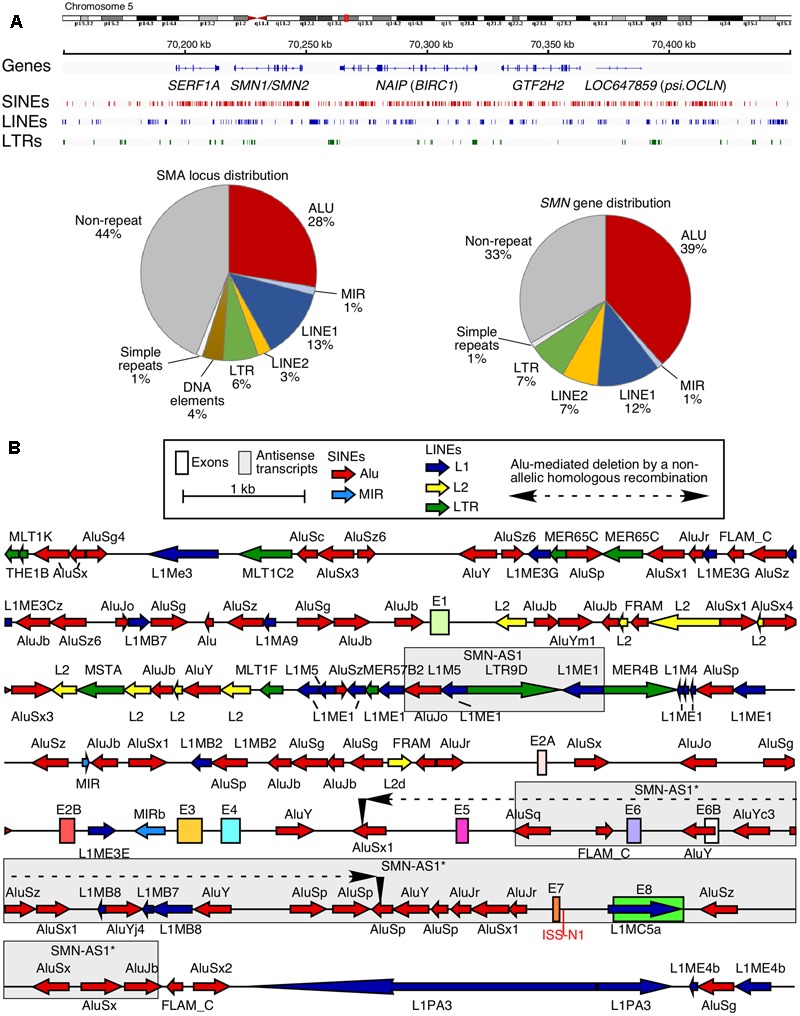
High prevalence of Alu-derived repeats in *SMN* locus. **(A)** Genomic overview of the duplicated genomic region in chromosome 5 encompassing the *SMN* genes. Upper panel indicates the location of genes and the three most prevalent types of repeats: SINEs, LINEs, and long terminal repeats (LTRs). Lower panel pie charts indicate the total percentage of sequence occupied by different types of repeats in either the whole SMN locus including other duplicated genes (left) or in the *SMN* gene itself (right). **(B)** Detailed view of the *SMN* gene and nearby surrounding sequences. *SMN* exons are indicated with colored boxes. Repeat sequences are indicated as colored arrows, with the direction of the arrow indicating the orientation of the repeat-derived sequence. An Alu-mediated recombination event which resulted in a deletion in an SMA patient (Wirth et al., 1999) is indicated. Two boxed regions indicate the location of known antisense transcripts derived from the *SMN* locus (d’Ydewalle et al., 2017; Woo et al., 2017).

Chromosome 5 is one of the largest human chromosomes and harbors at least ten clusters of intrachromosomal repeats (Schmutz et al., 2004). One such intrachromosomal repeat at the 5q13.3 locus resulted in the generation of two nearly identical copies of *Survival Motor Neuron* gene: *SMN1* and *SMN2* (Lefebvre et al., 1995; Schmutz et al., 2004). Other duplicate genes at this locus include *SERF1, NAIP* (*BIRC1*), and *LOC647859* (*psi.OCLN*) (Schmutz et al., 2004; **Figure 1A**). Alu elements occupy ∼28% of the sequence at the 5q13.3 locus and account for a whopping 39% of the sequence in the *SMN* genes (**Figure 1A**). However, very limited attention has been paid toward understanding the consequences of such a high abundance of Alu elements in the *SMN* genes. Both *SMN* genes contain nine exons and code for SMN, an essential protein involved in various processes including snRNP biogenesis, transcription, translation, selenoprotein synthesis, stress granule formation, signal recognition particle biogenesis, signal transduction, vesicular transport, and motor neuron trafficking (Singh et al., 2017c). While the coding region of *SMN* is conserved between human and rodents, there are substantial differences in the promoter, intronic, the 5′ and 3′ untranslated regions (UTRs) primarily due to insertion of TEs including Alu-like sequences (**Figure 1B**). *SMN1* and *SMN2* differ in how the last coding exon, exon 7, is spliced (Singh, 2007; Singh and Singh, 2011; Singh et al., 2015a, 2017c). In the case of *SMN1*, all nine exons are included to produce the full-length transcript coding for the full-length SMN. In the case of *SMN2*, the majority of transcripts lack exon 7 due to a critical C-to-T mutation at the 6th position (C6U) of exon 7 (Lorson et al., 1999; Monani et al., 1999a). Transcripts lacking exon 7 code for SMNΔ7, an unstable protein, which is only partially functional (Lorson et al., 1998; Cho and Dreyfuss, 2010). Loss of *SMN1* leads to an SMN deficit, resulting in spinal muscular atrophy (SMA), a devastating genetic disease of children and infants (Ahmad et al., 2016). Among various options for SMA therapy, correction of *SMN2* exon 7 splicing has shown high promise (Seo et al., 2013; Howell et al., 2014). The recently approved drug Spinraza^TM^ (nusinersen) for SMA is an antisense oligonucleotide (ASO) that fully corrects *SMN2* exon 7 splicing upon sequestering intronic splicing silencer N1 (ISS-N1) located within intron 7 (**Figure 1B**; Singh et al., 2006, 2017b; Ottesen, 2017). Small ASOs targeting a GC-rich sequence overlapping ISS-N1 also promote *SMN2* exon 7 inclusion and provide therapeutic benefits in mouse models of SMA (Singh et al., 2009, 2015b; Sivanesan et al., 2013; Kiel et al., 2014). We have recently shown that ISS-N1 sequesters a cryptic 5′ss, activation of which carries therapeutic implications for patients who cannot be treated by Spinraza^TM^ or any other ASO targeting ISS-N1 (Singh et al., 2017a). In addition to SMA, SMN has been found to play an important role in male reproductive organ development and male fertility in mammals (Ottesen et al., 2016). Aberrant expression and/or localization of SMN have also been associated with other human diseases, including amyotrophic lateral sclerosis, inclusion body myositis and osteoarthritis (Singh et al., 2017c). Considering that Alu elements affect multiple steps of gene expression, understanding their potential role in the regulation of expression of the disease-linked *SMN* gene has broad implications.

Given the high abundance of Alu elements within the introns of both *SMN* genes, one would expect exonization of one or several of Alu elements. However, until recently, an exonized Alu element of *SMN* evaded detection due in part to the lack of an appropriate assay. We optimized a multi-exon-skipping-detection assay (MESDA) that determines the relative abundance of all *SMN* splice-isoforms in a single reaction (Singh et al., 2012; Seo et al., 2016a,b). Using MESDA, we detected a novel exon, exon 6B, generated by exonization of an Alu element within *SMN* intron 6 (Seo et al., 2016a). Others have independently validated/identified the exon 6B-containing transcripts in various human tissues and cell lines (Yoshimoto et al., 2016; Sutherland et al., 2017). In this brief review, we describe the likely impact of Alu elements on expression of SMN. We also discuss the possible mechanism(s) of regulation of *SMN* exon 6B splicing and potential consequences of the generation of the exon 6B-containing transcripts.

## Alu Elements and Pathogenesis of SMA

Transposable elements, including Alu elements, occupy more than 65% of the human *SMN* (*SMN1* or *SMN2*) gene that spans ∼44 kb sequence including a ∼10 kb promoter region (**Figure 1**). Such a dense distribution of intrachromosomal repetitive Alu elements is often associated with NAHR to repair double strand breaks. Alu elements also serve as hotspots for non-homologous end joining (NHEJ)-based DNA repairs. Both NAHR and NHEJ that involve intrachromosomal Alu repeats potentially result in deletion or duplication of sequences ranging in size from 300 bases to tens of kilobases (Sen et al., 2006). A vast majority of SMA cases arise from deletion of a short genomic sequence encompassing exons 7 and 8 of *SMN1* (Lefebvre et al., 1995). Although the information of the exact breakpoints of these deletions is not publicly available, they appear to include the Alu-rich intron 6 and the Alu-rich intergenic region downstream of exon 8. Interestingly, an AluSx1 and an AluSz are located immediately upstream of exon 7 and downstream of exon 8, respectively. These two Alu elements are known to be involved in the deletion in *MLL* gene associated with leukemia and cell-based experiments confirm that both NAHR- and NHEJ-based DNA repair mechanisms are the potential mechanisms of DNA deletion (Morales et al., 2015). Hence, it is likely that the pathogenic deletion of exons 7 and 8 of *SMN1* also happens through both mechanisms. Other SMA cases involve Alu/Alu-mediated deletion of sequences from intron 4 through intron 6 (**Figure 1B**; Wirth et al., 1999). The breakpoint of this Alu/Alu-mediated deletion occurred in the first 100 bp of the Alu elements. Such breakpoints are common characteristics of NAHR-mediated deletion as recently confirmed by a novel cell-based reporter system (Morales et al., 2015). While most SMA cases arise from inheritance from unaffected carriers, ∼2% of patients acquire *de novo* mutations (Wirth et al., 1997). It is likely that Alu elements have contributed toward *de novo* mutations in *SMN1* through NAHR- and/or NHEJ-based repair mechanisms in germline or in progenitors of germline cells.

## Alu Elements and *SMN* Transcription

**FIGURE 2:**
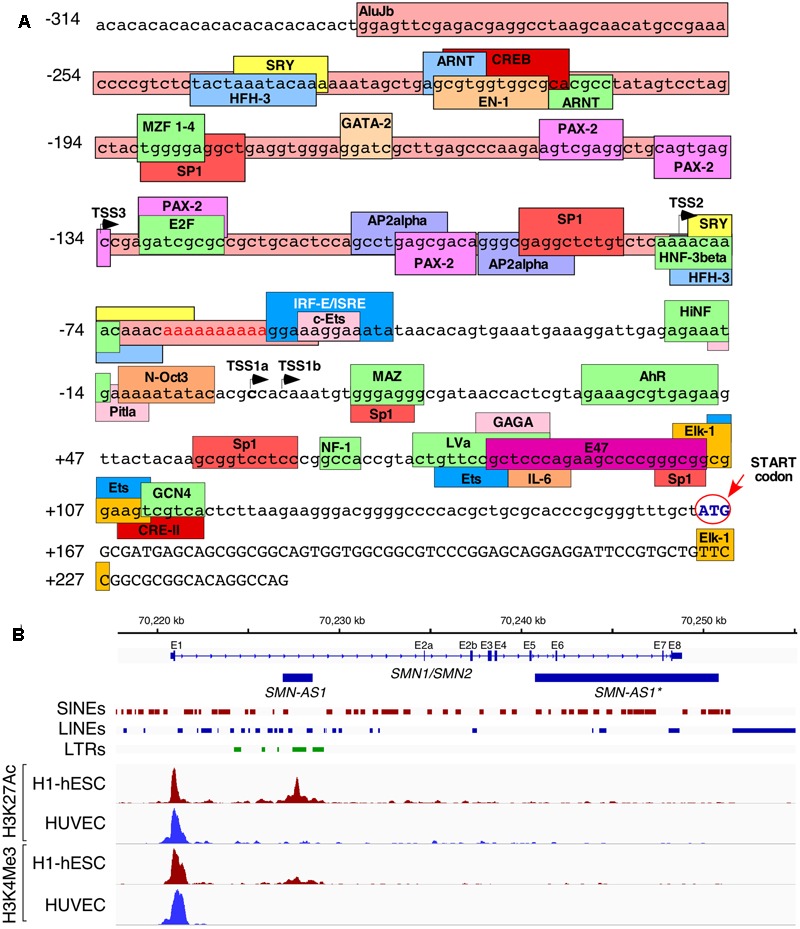
Alu repeats reshape transcriptional regulation of *SMN*. **(A)** Overview of the promoter region of *SMN*. Lowercase letters indicate untranscribed or untranslated regions, uppercase letters indicate coding sequences. A partial AluJb element is inserted from 289 to 54 bases upstream of the canonical TSS (TSS1a) and is indicated with a dark pink colored box. Other colored boxes indicate the locations of putative promoter elements and/or transcription factor binding sites. TSSs are indicated with black arrows. The start codon is written in blue and is indicated with a red arrow and circle. Promoter elements were either described previously (Singh et al., 2016) or were computationally predicted by ConSite (consite.genereg.net). **(B)** Locations of Polycomb-associated antisense transcripts derived from *SMN*. A genomic view of the *SMN* gene is shown. Location of *SMN* exons (E1–E8) and antisense transcripts are indicated with blue boxes, introns are indicated by lines with arrows indicating the direction of transcription. The locations of SINEs, LINEs, and LTRs are indicated. Four ChIP-Seq outputs are given below. The red peaks indicate the read depth from ChIP-Seq of human embryonic stem cells using antibodies against acetylated H3K27 (upper) or tri-methylated H3K4 (lower). The blue peaks indicate the same readout from umbilical cord-derived HUVEC cells. ChIP-Seq data was obtained from ENCODE and previously described by d’Ydewalle et al. (2017).

Alu elements have drastically impacted the epigenetic landscape of the human genome and have consequently contributed toward the unique regulation of transcription of human genes (Daniel et al., 2014; Tajaddod et al., 2016). When it comes to *SMN* genes, several lines of evidence support a strong effect of Alu-derived motifs on their transcription. For instance, a deletion of ∼1.1 kb sequence within the *SMN* promoter region containing several Alu elements produced more than fivefold increase in the transcription activity in a cell-based reporter assay (Echaniz-Laguna et al., 1999). Further, an AluJb located immediately upstream of the most frequently used transcription start site (TSS1a) harbors several transcription regulatory motifs including a fetal transcription start site, TSS2 (Germain-Desprez et al., 2001; **Figure 2A**). Another transcription start site, TSS3, located 134 bp upstream of TSS1a also falls within the AluJb sequence (Monani et al., 1999b). *SMN* transcripts generated from either TSS2 or TSS3 possess longer 5′UTR with significance to unique regulation of transport, stability, and translation of these mRNAs. Transcription regulatory motifs located within the Alu elements include AP2alpha, ARNT, CREB, E2F, EN-1, Ets, HNF-3beta, interferon-stimulated responsive element (ISRE), MZF 1-4, PAX-2, SP-1, and SRY (**Figure 2A**). However, the significance of these motifs remains to be investigated. Interestingly, promoters harboring Alu elements are subject to regulation by long-noncoding RNAs (lncRNAs). For instance, *ANRIL*, a lncRNA identified in a GWAS as a risk factor in coronary artery disease, has been suggested to regulate expression of a network of genes through Alu elements located in their promoters (Holdt et al., 2013). This lncRNA potentially recruits PRC2, the chromatin remodeling complex (Holdt et al., 2013). Interestingly, *SMN* locus has been shown to generate two lncRNAs through transcription in the antisense direction. One of these lncRNAs termed *SMN-AS1* is a ∼1.6 kb long transcript that maps to the Alu-rich intron 1 (d’Ydewalle et al., 2017; **Figure 2B**). The other lncRNA termed *SMN-AS1^∗^* is a ∼10 kb long transcript, which maps to the Alu-rich regions that contains a portion of intron 5, intron 6 and the intergenic region downstream of exon 8 (Woo et al., 2017; **Figure 2B**). Depletion of *SMN-AS1* or *SMN-AS1*^∗^ has been found to enhance transcription of *SMN2* (d’Ydewalle et al., 2017; Woo et al., 2017). Interestingly, *SMN-AS1* maps to a chromatin region rich in acetylated and/or methylated histone H3 in embryonic stem cells, suggesting a tissue-specific regulation of transcription by this lncRNA (**Figure 2B**). In contrast, *SMN-AS1*^∗^ is expressed from a region which is not so rich in histone H3 modifications (**Figure 2B**). It has been proposed that both *SMN-AS1* and *SMN-AS1^∗^* modulate rate of transcription elongation through recruitment of the PRC2 complex (d’Ydewalle et al., 2017; Woo et al., 2017).

## Alu Elements and *SMN* Splicing

**FIGURE 3:**
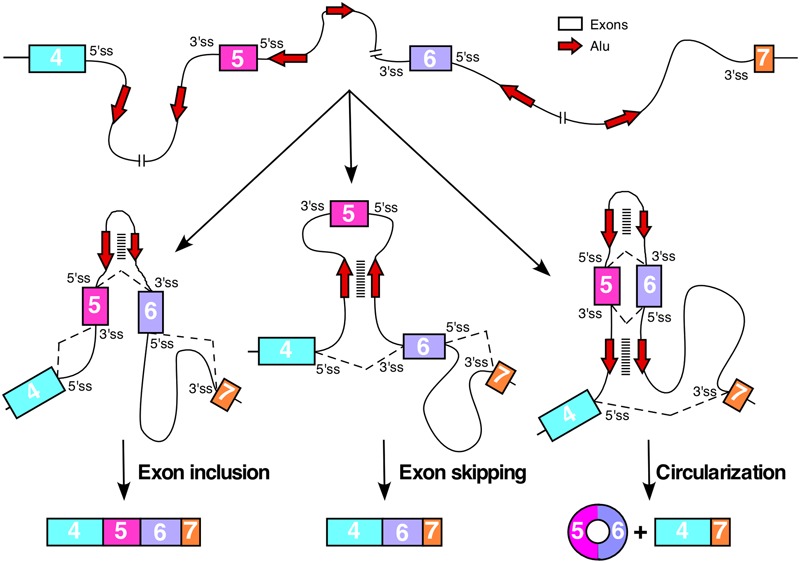
Consequences of Alu–Alu base pairing within *SMN* pre-mRNA. Diagrammatic representation of the partial *SMN* pre-mRNA (not to the scale) showing inclusion, skipping and circularization of exon 5. Exons are shown in colored boxes, whereas, red arrows indicated Alu elements. For simplicity, only two Alu elements per intron are shown. Base pairing between complementary Alu sequences are shown by stacked lines. Various hypothetical scenarios of splicing reactions involving different combinations of splice site pairings are shown by broken lines. Base pairing between Alu sequences within intron 5 promotes inclusion of exon 5, whereas, base pairing between intronic Alu sequences flanking exon 5 promotes exon 5 skipping. Base pairing between Alu elements within intron 5 combined with the base pairing between intronic Alu sequences upstream of exon 5 and downstream of exon 6 promotes circularization event.

Currently there is no study on the impact of Alu elements on splicing of various *SMN* exons. Alu elements can affect pre-mRNA splicing depending upon their sequence, orientation, location, and abundance. When present as inverted repeats, Alu elements form long double-stranded structures looping out intronic and/or exonic sequences. In cases where intra-intronic sequences are looped out, the 5′ and the 3′ ss are brought into close proximity, favoring intron removal (**Figure 3**). However, when an exon is looped out, its skipping is likely to be favored due to sequestration of the splice sites in the loop and increased competition from upstream and downstream splice sites, which are now in closer proximity to each other (**Figure 3**). The high abundance of Alu elements in *SMN* introns serve as the potential source for the intragenic base pairing among Alu sequences with opposite orientations. It is not known if some of these Alu-associated intra-intronic structures of *SMN* pre-mRNA are stabilized by ADARs. Several *SMN* exons are susceptible to skipping under conditions of oxidative stress (Singh et al., 2012; Seo et al., 2016b). It is likely that the ATP deficit caused by oxidative stress reduces the efficiency of RNA helicases such as DHX9, which unwinds the Alu-associated secondary structures within *SMN* pre-mRNA.

In addition to secondary-structure-associated regulation of splicing, Alu sequences can also recruit splicing factors on pre-mRNAs by interacting with complementary Alu sequences in lncRNAs. One such interaction has recently been proposed for 5S-OT, a lncRNA transcribed from 5S ribosomal RNA gene (Hu et al., 2016). The 3′-end of 5S-OT contains an Alu-derived 152 nt sequence that is complementary to the 3′ region of the sense Alu elements within introns 1, 2b, 4, and 6 of *SMN*. A polypyrimidine tract (Py) in the middle of the 5S-OT recruits the splicing factor U2AF65. Bioinformatics analysis revealed that 5S-OT regulates splicing of several exons, for which the Alu elements are located in the sense direction within the 2-kb upstream and/or downstream (Hu et al., 2016). In particular, knockdown of 5S-OT promoted inclusion and skipping of exons that had Alu elements located downstream and upstream of these exon, respectively. We speculate that splicing of *SMN* exons are regulated by 5S-OT, *SMN-AS1, SMN-AS1^∗^*, and possibly several other Alu-containing lncRNAs.

## Generation of Circular RNAs by *SMN* Genes

Circular RNAs (circRNAs) are generated by back splicing in which the 5′ss of an exon joins the 3′ss of an upstream exon. In agreement with the potential link between Alu elements and back splicing, Alu elements are highly enriched upstream and downstream of splice sites associated with circRNA formation (Jeck et al., 2013). Based on the online repository circBase, at least two circRNAs are generated by *SMN* (Glazar et al., 2014). One of these circRNAs is generated by back splicing of the 5′ss of exon 4 with the 3′ss of exon 2B, whereas, the other circRNA is generated by back splicing of the 5′ss of exon 6 with the 3′ss of the exon 5 (**Figure 3**). Based on the high density of intronic Alu elements, we expect generation of additional circRNAs by *SMN* genes. Owing to their extreme stability, even small levels of circRNAs may affect cellular metabolism by sequestering miRNAs and regulatory RNA-binding proteins (Hansen et al., 2013; Memczak et al., 2013). We expect that various *SMN* circRNAs are differentially expressed in different tissues. Future studies will determine what circRNAs are generated by *SMN* genes and how they impact the formation of linear *SMN* transcripts and affects cellular metabolism in different tissues. It will be also important to know if differential splicing of exon 7 leads to distinct circRNA patterns of *SMN1* and *SMN2*.

## Exonization of an Intronic Alu Element

**FIGURE 4:**
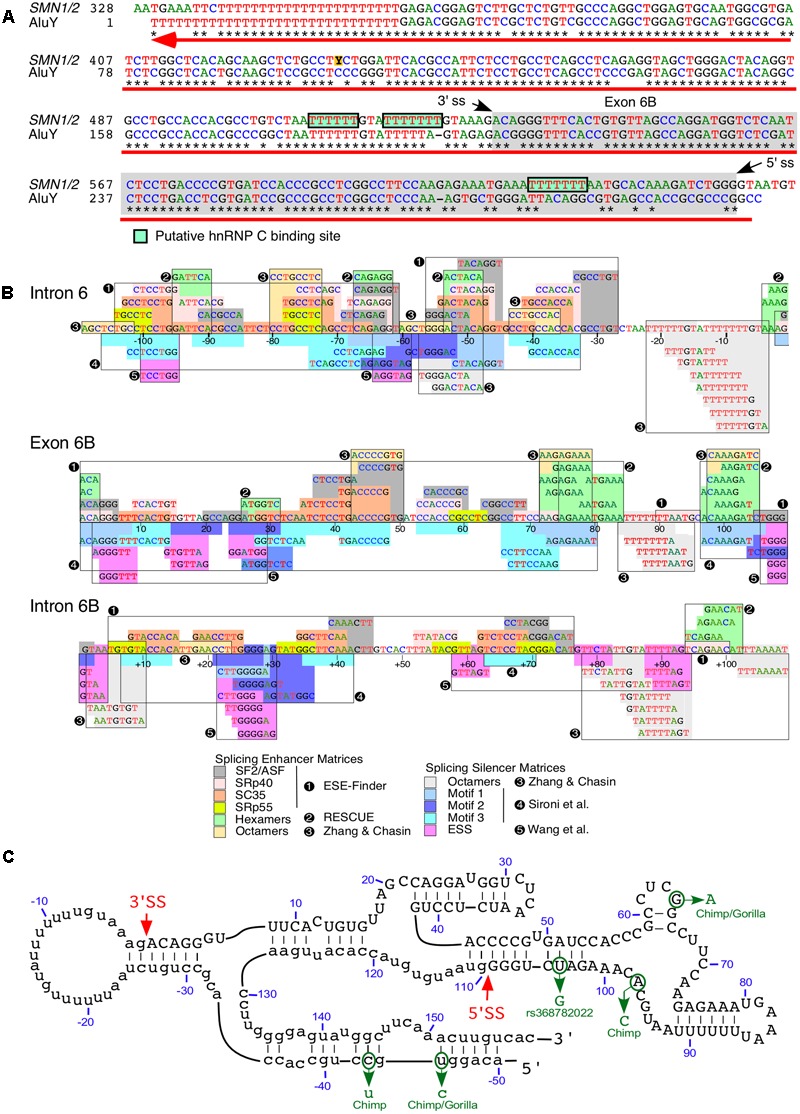
Exon 6B is derived from an intronic Alu element. **(A)** Alignment of SMN intron 6 region spanning exon 6B. Numbering starts from the beginning of intron 6. Stars signify sequence identity. Hyphens designate the positions where gaps were introduced to maximize sequence identity. The gray box indicates exon 6B sequences and the green boxes indicate putative binding sites for hnRNP C. The black arrows indicate splice site (ss) positions of exon 6B. The red arrow indicates position and direction of AluY insertion (reverse and complement) which are obtained from Dfam (Accession: DF0000002). **(B)** Predicted splicing cis-elements. The exon 6B and 109 nt of upstream and downstream intronic sequences are shown. Colored boxes indicate potential regulatory elements identified by Human Splicing Finder (Desmet et al., 2009). Potential splicing enhancers are indicated above the *SMN* sequence and splicing silencers are below. Color code is explained in the bottom panel, where numbers indicate the software tool used for identification or publications in which motifs were originally described. Exonic splicing enhancer (ESE) finder is described in (Cartegni et al., 2003). RESCUE refers to an algorithm that predicts ESEs (Fairbrother et al., 2002). Octamer motifs are described in (Zhang and Chasin, 2004). Motifs 1-3 are described in (Sironi et al., 2004). Silencer motifs highlighted in pink are described in (Wang et al., 2004). **(C)** Secondary structure of *SMN* exon 6B. Numbering starts from the beginning of exon 6B. Exon 6B sequences are shown in capital letters, while adjacent intronic sequences are shown in lower-case letters. The red arrows indicate ss positions of exon 6B. The green arrows indicate sequence differences of exon 6B between human and primates. The secondary structure was predicted using mfold algorithm (Zuker, 2003).

We recently reported a novel exon, exon 6B, generated by exonization of a 109-nt long sequence located within *SMN* intron 6 (Seo et al., 2016a; **Figure 4A**). Thus far, exon 6B is the only known exon to be derived from an Alu element within *SMN*. Exon 6B maps to the left arm of the antisense sequence of an Alu element and appears to be conserved in all members of the Hominidae family (Seo et al., 2016a; **Figure 4A**). Considering most Alu-derived exons originate from the right arm of the antisense sequence of an Alu element (Sorek et al., 2002), generation of exon 6B is an example of a rare event. The relative abundance of exon 6B-containing transcripts was found to be low compared to transcripts lacking exon 6B. This is in part due to degradation of exon 6B-containing transcripts by NMD, a translation dependent process. Consistently, inhibition of translation by cycloheximide elevated the levels of exon 6B-containing transcripts (Seo et al., 2016a). Depletion of UPF1, an essential component of NMD pathway, was also found to upregulate exon 6B-containing transcripts. Degradation of exon 6B-containing transcripts could also be facilitated by SMD, an UPF1-dependent process triggered by base pairing of Alu sequences in mRNAs with the Alu-containing lncRNAs (Park and Maquat, 2013).

Consistent with the low abundance of exon 6B-containing transcripts, the predicted strengths of splice sites of exon 6B were significantly lower than that for the neighboring exons 6 and 7 (Seo et al., 2016a). However, we observed a dense map of overlapping enhancer motifs within exon 6B and its flanking intronic sequences (**Figure 4B**). Of note, nucleotide differences between exon 6B and AluY are predicted to create several enhancer elements toward the 3′ end of this exon (**Figure 4B**). These elements might contribute to the regulation of exon 6B splicing. As expected, the incorporation of exon 6B appeared to be suppressed by hnRNP C that is known to inhibit the exonization of intronic Alu elements (Seo et al., 2016a). It has been demonstrated that TIA1 regulates *SMN* exon 7 splicing through interaction with the intronic uridine-rich clusters downstream of exon 7 (Singh et al., 2011). Interestingly, similar uridine-rich clusters are present downstream of exon 6B, pointing to the potential involvement of TIA1 in splicing of exon 6B. However, analysis of the publicly available transcriptome data showed no effect of TIA1 depletion on splicing of exon 6B (Seo et al., 2016a). Consistent with these results, we did not detect changes in the splicing of *SMN2* exon 6B in a SMA mouse model in which *Tia1* was deleted (Howell et al., 2017b).

We have previously shown that RNA secondary structures that sequester the splice sites affect inclusion of *SMN* exon 7 (Singh et al., 2004a,b,c, 2007, 2013). Interestingly, the predicted secondary structure of exon 6B and its flanking intronic sequences puts both the 3′ss and the 5′ss in stems (**Figure 4C**). It is likely that these stems play a negative role in inclusion of exon 6B by suppressing the splice site recognition. We also observed that the most of the exon 6B sequence is sequestered within the predicted terminal and internal stems (**Figure 4C**). It remains to be seen if these intra-exonic structures play any role in exon 6B splicing regulation. Critical role of an intra-intronic structure formed by a unique long-distance interaction has been demonstrated for regulation of *SMN* exon 7 splicing (Singh et al., 2010, 2013; Howell et al., 2017a). Interestingly, the predicted secondary structure of sequences downstream of exon 6B reveal long-distance interactions formed between sense and antisense Alu elements (not shown). It is likely that these structures play some role in regulation of exon 6B splicing.

## Potential Functions of SMN6B

**FIGURE 5:**
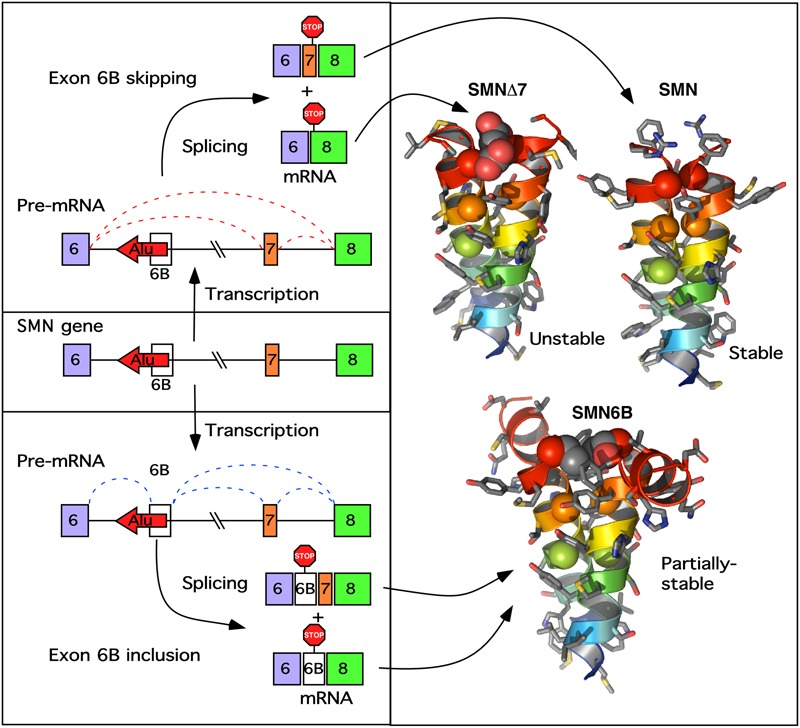
A model of exon 6B action. **(Left)** Describes the transcription of the *SMN* gene and pre-mRNA splicing producing either the 6B-skipped (upper) or 6B-included (lower mRNA). Exons are indicated as colored boxes, the Alu element from which exon 6B is derived is indicated as a red arrow, introns are shown as lines. Potential splicing events are shown as red (exon 6B-skipped) or blue (exon 6B-included) dotted lines. Locations of stop codons generated by each potential transcript are indicated. **(Right)** Shows the computationally predicted glycine zipper dimers formed by the YG boxes at the C termini of each of the SMN protein isoforms. Both SMNΔ7 and SMN6B have altered YG boxes resulting in an increase in the inter-helical distances of the coiled-coil interaction, potentially reducing oligomerization. In SMNΔ7 this results in an unstable degron (Cho and Dreyfuss, 2010), whereas in SMN6B the destabilization is less pronounced (Seo et al., 2016a).

Exon 6B-containg transcripts are expressed in all tissues and code for SMN6B, which contains an identical number of amino acids as SMN (Seo et al., 2016a). However, SMN differs from SMN6B by sixteen C-terminal amino acids (**Figure 5**). In particular, in the transcripts containing exon 7 but lacking exon 6B, the last sixteen C-terminal amino acids are coded by exon 7. It is known that the amino acids coded by exon 7 play an important role in stabilization, self-oligomerization and protein-protein interactions (Singh et al., 2017c). Hence, the loss of these amino acids is the primary cause of the poor stability as well as the suboptimal functions of SMNΔ7 (Cho and Dreyfuss, 2010). A side-by-side comparison showed that SMN6B is less stable than SMN (Seo et al., 2016a). At the same time, the stability of SMN6B was found to be greater than SMNΔ7 (Seo et al., 2016a). Similar to SMN, SMN6B localizes to both the nucleus and the cytosol. Further, SMN6B interacts with Gemin2, which is associated with most SMN functions. Hence, it is likely that SMN and SMN6B share most of the cellular functions including snRNP biogenesis, transcription, translation, macromolecular trafficking, telomerase biogenesis, selenoproteins biosynthesis, signal transduction and stress granule formation. High copy numbers of *SMN2* are associated with low severity of SMA, likely due to the expected high levels of SMN6B. However, levels of SMN6B produced in SMA patients remain unknown. Factors that regulate expression of SMN6B are expected to modulate the severity of SMA. Future studies will determine if SMN6B has a tissue-specific function. Of note, production of SMN6B will confer unparalleled therapeutic benefits in SMA patients carrying deletions of genomic sequences downstream of exon 6B. A proper understanding of inhibitory cis-elements that regulate exon 6B splicing will provide novel targets for the stimulation of exon 6B inclusion leading to the production of SMN6B.

## Concluding Remarks

Given the importance of SMN in cellular metabolism and its association with various pathological conditions, there has been general interest in the mechanisms by which levels of SMN are regulated. Since the evolution of primates, the genomic landscape of the *SMN* locus has undergone massive changes, including duplication sometime before the divergence of human and chimpanzee lineages and the human-specific mutations characteristic of *SMN2* (Rochette et al., 2001). Among these changes, and perhaps a driving force for other ones, are the insertion of a large number of Alu elements into intronic and intergenic regions (**Figure 1**). Based on the deletions in the Alu-rich promoter region as well as the recent discoveries of the Alu-containing lncRNAs, we propose that the regulation of transcription of the *SMN* genes is distinct in primates. Similarly, given the preponderance of Alu elements in most *SMN* introns, we anticipate that splicing of *SMN* exons is uniquely regulated in primates. There is a strong likelihood that human *SMN* genes are subjected to a unique transcription-coupled splicing regulation primarily due to the abundance of Alu elements within *SMN* genes. However, mechanism of such regulation remains to be investigated.

The finding of Alu-derived exon 6B adds an additional regulatory step in the expression of *SMN* genes. Our results suggest that inclusion of exon 6B inhibits skipping of *SMN2* exon 7 (Seo et al., 2016a). However, irrespective of exon 7 inclusion or skipping, transcripts harboring exon 6B code for the same SMN6B protein, which displays higher stability than SMNΔ7 (Seo et al., 2016a). Our findings suggest that an enhanced expression of SMN6B may confer therapeutic benefits when SMN is absent or expressed at very low levels. A handful of genes code for proteins with C-terminal sequences similar to those coded by exon 6B (Seo et al., 2016a). Hence, it will be interesting to know if these proteins possess some common properties. Mice carry B1 elements that share several properties with Alu elements. There is also evidence to suggest that several of the functions of Alu elements are carried by B1 elements in mice (Aktaş et al., 2017). However, due to a size difference between Alu and B1 elements, it is expected that B1 elements cannot perform all Alu-associated functions. In particular, it is highly unlikely that circRNAs induced by Alu elements are also generated in non-primates. Further, human *SMN* genes are unique in producing lncRNAs harboring Alu elements. Future studies will determine how insertion of Alu elements have impacted the regulation and regulatory activities of *SMN* genes, which are linked to various pathological conditions in humans.

## Author Contributions

EO, JS, and RS analyzed literature and/or publically available data. EO, JS, NS, and RS designed diagrams and wrote the manuscript.

## Conflict of Interest Statement

The authors declare that the research was conducted in the absence of any commercial or financial relationships that could be construed as a potential conflict of interest.
